# Intradermal Application of Allogenic Wharton’s Jelly Mesenchymal Stem Cells for Chronic Post-Thoracotomy Wound in an Elderly Patient After Coronary Artery Bypass Grafting: Clinical Case with Brief Literature Review

**DOI:** 10.3390/diseases14010027

**Published:** 2026-01-08

**Authors:** Anastassiya Ganina, Abay Baigenzhin, Elmira Chuvakova, Naizabek Yerzhigit, Anuar Zhunussov, Aizhan Akhayeva, Larissa Kozina, Oleg Lookin, Manarbek Askarov

**Affiliations:** JSC National Scientific Medical Center, 42 Abylay Khan Ave., Astana 010009, Kazakhstan; e.chuvakova@nnmc.kz (E.C.); naizabek1997@gmail.com (N.Y.); dr.anuar.zhunussov@mail.ru (A.Z.); akhaeva@mail.ru (A.A.); l.kozina@nnmc.kz (L.K.); lookinoleg@gmail.com (O.L.); askarova8109@mail.ru (M.A.)

**Keywords:** mesenchymal stem cells, Wharton’s jelly, allogenic transplantation, deep sternal wound infection, aged patient, regeneration

## Abstract

**Background:** Chronically non-healing thoracic wounds after cardiac and non-cardiac thoracotomy, including cases when coronary artery bypass grafting (CABG) is performed, represent a great clinical challenge. It is often that a conservative treatment of the wounds does not provide effective regeneration of the damaged tissues. It is especially critical in patients with infected wounds, in patients owning a systemic infection, and in elderly people. **Methods:** The article presents a case report of successful treatment of a 63-year-old man with refractory chronic osteomyelitis of the sternum and mediastinitis four years after CABG, complicated by COVID-19 at the time of reconstructive surgery. Due to the low effectiveness of conservative treatment methods, a two-stage approach was applied: radical surgical wound debridement followed by infiltration of the wound with allogenic mesenchymal stromal cells (MSCs) of Wharton’s jelly (WJ-MSCs). **Results:** This double-stage therapy successfully modulated the inflammatory environment and stimulated granulation, facilitating final thoracoplasty and osteosynthesis. The patient achieved complete healing of the sternum, demonstrating benefits of WJ-MSCs in treating conservative treatment-resistant infections in the surgical wound. **Conclusions:** The advantages of using perinatal mesenchymal stem cells, with WJ-MSCs as a type of this class of MSCs, were demonstrated in treating chronically infected sternal surgical wounds. We also compared their regenerative properties to other stem cell types like bone marrow MSCs.

## Simple Summary

Invasive thoracic surgery represents a great post-operative problem—a large and slowly healing surgical wound, often with infectious complications. In elderly people, this may turn out to be a severe chronic state resistant to the common treatment. We describe a clinical case of an elderly man with refractory chronic sternal osteomyelitis and mediastinitis developed after CABG that persisted for four years. As the conservative treatments—wound debridement and antibacterial therapy—were non-effective, the wound was subjected to the intradermal injections, three times in total, by Wharton’s jelly mesenchymal stromal cells. The outcome was the prominent recovery of wound state followed by complete wound healing. Our case demonstrates beneficial effects of WJ-MSCs in treating conservative treatment-resistant infections in the surgical wound, most likely due to modulation of inflammatory environment in the wound.

## 1. Introduction

In patients who have undergone median sternotomy, especially after coronary artery bypass grafting (CABG), deep sternal wound infection (DSWI) often develops into chronic sternal osteomyelitis, which represents a serious clinical complication [1,2]. Median sternotomy may be complicated by DSWI in 0.25% to 5% of patients [3]. Despite timely and comprehensive treatment, mortality in such patients remains shockingly high. Systematic reviews and meta-analyses show that DSWI can account for about half of in-hospital mortality. This is one of the most life-threatening complications that can occur during cardiac surgery [4].

Mesenchymal stem cells (MSCs) are a prospective option for treating chronic infections including DSWI. The secretome of MSCs has prominent antimicrobial properties [5]. The effect is achieved through the secretion of antimicrobial peptides, such as LL-37, which destroy bacterial membranes directly [6], and via powerful immunomodulation, which “reprograms” local immune cells, such as macrophages. Due to the MSCs’ activation, macrophages can switch from the pro-inflammatory phenotype M1 to the anti-inflammatory and phagocytic M2, which improves bacterial excretion and prevents destructive long-term inflammation [7,8]. The cell extracts obtained from MSCs have high regenerative capacity for skin wound healing via several pathways including reactivation of dermal fibroblasts [9]. In addition, MSCs themselves can differentiate to the connective tissue and replace other types of cells in the wound site, and they can potentiate angiogenesis and migration of cells to the wound site [10].

On the other hand, not all types of MSCs have equal efficiency and regenerative capacity. The perinatal MSCs, i.e., obtained from tissues that surround the fetus, have several distinct advantages over other types of “adult” MSCs: higher extent of immaturity, higher capacity for proliferation, and lower immunogenicity [11,12]. In addition, they exhibit low propensity to pro-tumor activity or even possess anti-tumor activity [13]. Furthermore, perinatal MSCs are collected from biological “waste” sources, which are typically discarded during labor: amniotic fluid, placenta, and umbilical cord. Therefore, these fluids and tissues represent the MSCs’ sources, which are free from ethical concerns.

Wharton’s jelly, which is the component of umbilical cord, is one of the generous sources for immature MSCs. The Wharton’s jelly MSCs (WJ-MSCs) are considered very potent cells in regenerative medicine due to their high capacity for paracrine regulation via secretion of many active factors [14]. WJ-MSCs effectively promote the growth of epithelial tissue and accelerate wound healing [15]. Their successful application was validated in deep skin wounds [16] and in infected wounds after severe burns [17]. Nevertheless, direct application of WJ-MSCs is not widely used and therefore their wound regeneration outcomes are insufficiently reported, especially in chronic states of infected surgical wounds in elderly people who have diminished cellular capacity of tissue reparation.

We describe here a case report of an aged man diagnosed with refractory chronic osteomyelitis of the sternum and mediastinitis developed after CABG. The presence of chronically non-healing wound and concomitant COVID-19 infection in the perioperative period required a review of the tactics by introducing a personalized multi-stage approach in order to obtain as beneficial effects as possible.

## 2. Case Presentation

A male, 63-year-old patient suffered from an acute myocardial infarction for the first time in 2014. Staged coronary artery stenting was performed. In 2016, the patient experienced severe angina again. According to the results of the control coronary angiography, a multivessel lesion of the coronary arteries was diagnosed. In December 2016, the patient underwent CABG under off-pump conditions (without extracorporeal blood circulation). The intervention also included mammary coronary bypass surgery of the anterior interventricular artery, as well as autovenous bypass surgery (using vena saphena magna) of the diagonal artery, obtuse edge branch from the circumflex artery, and the circumflex artery itself.

**Figure 1:**
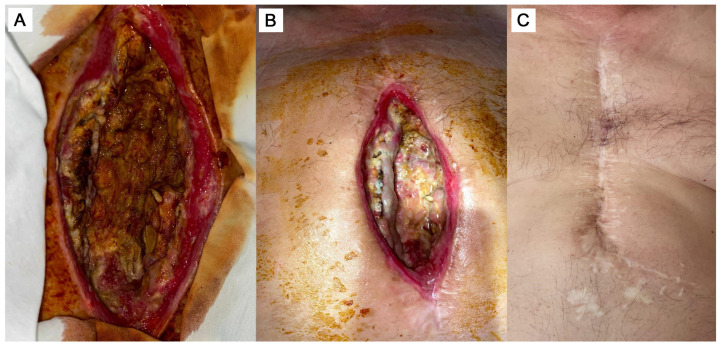
The state of the surgical wound before (**A**), 6 days after the commencing of cell product administration as an additive to the surgical correction (**B**), and 4 years after the treatment (**C**).

The patient started experiencing purulent discharge from the lower third of the postoperative sternum scar approximately three weeks post-CABG, since the beginning of 2017 (Figure 1A). In 2017, 2018, and 2020, three more excisions of the fistula were performed. Signs of prolonged inflammation, recurrent fistula formation, and lack of the wound healing persisted despite antibiotic therapy (bacterial infection by Staphylococcus aureus, ~10^5^ colony-forming units per milliliter, treated by Cefuroxime (0.75 g each 8 h), then Ceftriaxone (2 g each 12 h)).

On 11 May 2021, the patient underwent a surgical revision due to a significant increase in the systemic inflammatory response: C-reactive protein (CRP) = 168.46 mg/L, presepsin = 351 pg/mL, leukocytes = 18.7 × 10^9^/L. The fistula, which passed through the sternum and extended into the pericardial cavity and the right ventricle, was resected. However, weak wound healing and continuing signs of chronic inflammation were observed during the next two weeks. On 27 May 2021, after receiving the patient’s informed consent, the wound edges were infiltrated with an allogenic cellular product containing MSCs and fibroblasts. The source for the cell product was the umbilical cord provided by another institution (maternity hospital), not from commercially available sources. The cell product with a concentration of 1 × 10^6^ cells in 1 mL of saline (0.9% NaCl) was used for the procedure. The manipulation was performed using a 1.0 mL sterile insulin syringe with a thin needle, which made it possible to ensure accurate dosing and minimize injury to perivulary tissues. The drug was administered intradermally and subcutaneously strictly along the perimeter of the wound, with an indentation of 0.5–1.0 cm from the edge of the defect. This procedure was repeated three times with an interval of 48 h (on days 1, 3, and 5) to ensure sustained therapeutic effect. The injection technique consisted of creating papules by injecting 0.1–0.2 mL into each point with 1.0 cm step between injections and with 10 to 15 injections in total. The injections were performed around the fistula to stimulate angiogenesis and accelerate marginal epithelialization before the planned thoracoplasty. The detailed protocol of culturing the allogenic cell product and measuring cell growth kinetics and cell count can be found in Supplement Materials (Table S1). The phenotypic features of the cultured cells, as obtained by flow cytometry using specific fluorescent markers, are described in Supplement Material as well (please see Figures S1–S3). During the following days, positive changes in the wound site were noted, i.e., cessation of pathological secretions and wound healing (Figure 1B).

At the stage of preparation for subsequent surgical treatment, a screening examination revealed the presence of SARS-CoV-2 virus RNA. The viral state was asymptomatic and had no significant effect on the healing of the postoperative wound. Before surgical correction as well as after reconstructive surgery, the patient was treated by systemic infusion of piperacillin + tazobactam (4.5 g each 8 h).

On 12 July 2021, local examination showed that the wound remained open. High systemic inflammation persisted at the time (CRP = 130.88 mg/L, erythrocyte sedimentation rate = 63 mm/h). EchoCG showed a decrease in the left ventricular ejection fraction (LVEF) to 49–50% and signs of left ventricular aneurysm. On 16 July 2021, final reconstruction operations were performed: osteosynthesis of the sternum using metal structures, autoplasty of the sternum using bone tissue, and rib refixation to the sternum. At the time of the intervention, the bottom of the wound was granular without any pathological discharge. The edges of the sternum and 5th, 6th, and 7th ribs were mobilized. A bone autograft was taken from the iliac crest and attached to the sternum defect with two metal plates. The graft was fixed on the cartilaginous joints of 5th, 6th, and 7th ribs.

After the surgery, the inflammation level gradually decreased (CRP to 47.31 mg/L by 27 July 2021). On 19 July 2021, the retrosternal drainage was removed for three days. The control EchoCG performed on 22 July 2021 showed that LVEF decreased to 39%. Despite this instrumental finding, the patient remained hemodynamically stable, with no signs of congestion or peripheral edema. Heart failure was effectively managed with guideline-directed medical therapy, including beta-blockers (bisoprolol), ACE inhibitors (lisinopril), and aldosterone antagonists. Consequently, the patient was discharged on 2 August 2021, in satisfactory condition after 21 days being in-hospital setting.

After discharge, the patient was under outpatient supervision. The sternum remained stable; the wound healed with primary tension. The improvement of general well-being, reduction in local signs of inflammation, and normalization of leukocytes and CRP levels were noted. A laboratory test one month after reconstructive surgery showed the following: CRP = 15.15 mg/L, leukocytes—10.66 × 10^9^/L. During the long-term follow-up, the state of the wound gradually improved, attaining full closure and complete regeneration of tissue. The state of the surgical wound approximately 4 years after the cell therapy is shown on Figure 1C.

## 3. Discussion

Experimental results obtained over the past decade show that human perinatal tissues such as the placenta, membranes, and umbilical cord, as well as perinatal fluids such as amniotic fluid and umbilical blood, contain various multipotent progenitor cells [18]. These populations of mesenchymal stem cells, also known as perinatal mesenchymal stem cells (P-MSC), are of great importance for regenerative medicine as they have several key advantages compared to “adult” types of MSCs (collectively A-MSC) like from bone marrow (BM-MSC) or adipose tissue (AD-MSC).

First, P-MSCs have significant regenerative potential because of a more naive and less differentiated phenotype (i.e., possess higher stemness) [19,20]. This is vital for clinical use, since hundreds of millions of cells are often required to achieve a therapeutic effect [21]. Next, P-MSCs are low-immunogenic and therefore a high number of successful cases of transplantation without rejection have been reported [22]. They poorly express class I major histocompatibility complex (MHC) molecules, as well as class II MHC molecules and co-stimulating molecules such as CD80 and CD86 [23]. This “immunoprivilege” allows them to be used in allogenic (donor) situations without strict requirements for suitable Human Leukocyte Antigen (HLA) [24]. Third, P-MSCs have a low tumorigenicity profile [25] or even exhibit anti-tumor properties [13]. Also, the cells carry fewer accumulated genetic mutations compared to A-MSCs, and this reduces the likelihood of oncogenic transformation in vitro [26,27]. Finally, perinatal tissues such as the umbilical cord and placenta are usually disposed of as medical waste after childbirth [18]. Therefore, collecting the material is completely safe and easily accessible [19]. Unlike other allogenic MSCs, which require invasive donor intervention, or embryonic stem cells, which are associated with intractable ethical problems, the collection of perinatal tissue does not raise ethical objections [22]. Table 1 comparatively summarizes the key properties and characteristics of P-MSCs and A-MSCs (BM-MSCs in this comparison).

**Figure 2:**
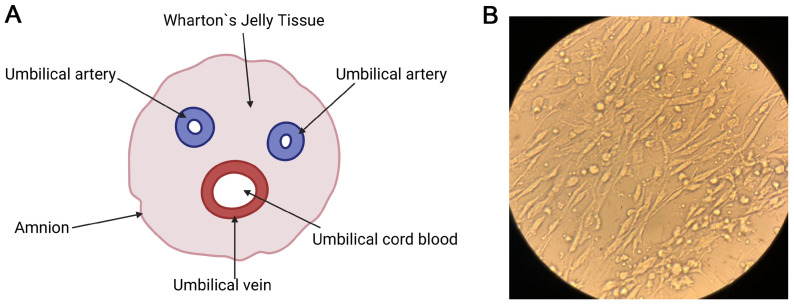
Mesenchymal stem cells obtained from Wharton’s jelly (WJ-MSCs). (**A**) Schematic representation of the human umbilical cord cross-section with main anatomical structures: the external amniotic membrane (amnion), which limits the Wharton’s jelly tissue (the mucous connective tissue), the vascular component includes two umbilical arteries (umbilical artery), and one umbilical vein (umbilical vein) containing umbilical cord blood. (**B**) The morphology of the WJ-MSCs cultured in vitro. The bright-field microscopic image shows the adhesive cells of a characteristic fusiform (fibroblast-like) shape with clear boundaries and pronounced processes (40×). The image in Panel (**A**) was created in Biorender. Naizabek Yerzhigit (2025) https://app.biorender.com/illustrations/6912c873d798ed7e2a9f80f3, accessed on 12 December 2025. The image in Panel (**B**) was obtained in our lab.

Wharton’s jelly mesenchymal stem cells (WJ-MSCs) are one of the subtypes of cells derived from perinatal tissues. Wharton’s jelly is a special type of connective tissue surrounding the umbilical cord vessels to provide their mechanical protection (Figure 2A). In addition, it is an important reservoir of stem cells, which is actually the “gold standard” for all P-MSCs [12,22]. WJ-MSCs exhibit the classical fibroblast shape when cultured in vitro (Figure 2B) and express standard markers defined by the International Society for Cell and Gene Therapy (ICST): they are positive for CD90, CD105, and CD73, and negative for hematopoietic markers such as CD45 and CD34 [12,28,29]. WJ-MSCs are characterized by a high rate of in vitro expansion, which makes it possible to obtain a therapeutically significant number of cells from a single donor sample in a short period of time [20]. The cells demonstrate the ability to differentiate in vitro into chondrocytes (cartilage tissue), osteocytes (bone tissue), and adipocytes (adipose tissue) [30,31]. Finally, immunomodulation is perhaps the most important therapeutic characteristic of WJ-MSC: they are not only “immunoprivileged”, but also affect the immune system [22,32]. They form a powerful anti-inflammatory environment by suppressing the proliferation of T-lymphocytes and regulating the function of B cells, dendritic cells, and natural killer NK-cells [30].

Clinical translation of MSC therapy for chronic wounds, bone infections, or chronic pain is generally characterized by inconsistent outcomes [33]. An illustrative example is a large double-blind randomized clinical trial, in which 114 patients with chronic low back pain received either allogeneic BM-MSC or placebo (sham) [34]. After 12 months, this study had not reached its primary endpoint, showing no statistically significant difference between the groups. Furthermore, a review of 449 clinical trials of MSCs in orthopedics (up to December 2023) revealed that only 12.5% of them published peer-reviewed results [35]. Importantly, more than half of the study protocols did not indicate the concentration of injected cells. Such methodological inconsistencies make it impossible to adequately compare and reproduce the results, hindering the development of MSC as a reliable therapeutic product [36]. On the other hand, the use of MSCs in wound healing therapy was confirmed to be efficient in wound closing, tissue reparation, and formation of new vessels, according to a meta-analysis based on over 30 studies with approximately 2500 patients; the most used MSCs were bone marrow and adipose tissue, i.e., “adult” types [5].

While many studies used adult MSCs (BM-MSCs or AD-MSCs), these cells have some limitations. First, despite BM-MSCs autografts allow for reducing immunological risks, bone marrow extraction from the iliac crest remains a significantly invasive and painful procedure that requires anesthesia and carries a risk of infection at the puncture site [37]. Secondly (most critical point), the yield of MSCs from bone marrow is extremely low: they account for only 0.001% to 0.01% of the total population of mononuclear cells [38]. This corresponds to the release of only 60–600 cells per milliliter of aspirate [37,39]. Such a minuscule amount makes direct therapeutic use impossible and requires significant ex vivo expansion to achieve therapeutic doses, i.e., millions of cells. In turn, this generates the intractable problem of replicative aging in vitro [40]. However, the most fundamental limitation, especially for autologous therapy, is biological aging in vivo. The functional characteristics of BM-MSC, including their proliferative and osteogenic potential, significantly decrease with age [41,42]. This is confirmed at the molecular level: donors aged over 60 years have a 3-fold decrease in mRNA expression of alkaline phosphatase—a key marker of osteogenesis—compared to donors younger than 50 years [43]. Thus, the clinical experience of using “adult” MSCs like BM-MSCs has revealed a therapeutic paradox: their autologous use is least effective in those who need it most (elderly patients).

Perinatal MSCs like WJ-MSCs are devoid of these key limitations [20]. The introduction of WJ-MSCs into the bone marrow of mice effectively promoted bone formation, exceeding the “gold standard” of BM-MSCs—62.5% of cases in WJ-MSCs vs. 25% in BM-MSCs [44]. This indicates that WJ-MSCs can actively participate in the process of regeneration of the sternum bone. In addition, both high viability of WJ-MSCs and their direct biological superiority over BM-MSCs in osteogenesis in vivo were shown [44]. Furthermore, the effective application of WJ-MSCs and/or their secretome in other chronic non-healing conditions like ulcer trophic wounds has been reported. For example, WJ-MSC secretome embedded in a gel-like substance for topical application can substantially relieve chronic wound condition [45]. Other recent randomized controlled clinical trials confirmed that the recovery from chronic diabetic ulcer occurred much faster if the WJ-MSC secretome/exosomes are applied superficially to the wound [46,47]. Infiltration of the wound defect with WJ-MSC significantly modulates local inflammatory response, reduces microbial load, and stimulates formation of adequate granulation tissue, creating favorable conditions for subsequent thoracoplasty and restoration of the anatomical integrity of the sternum [48,49,50].

The combination of advantages—non-invasiveness of collection, high proliferative potential, no donor age-related issues, prominent osteogenic activity, and effectiveness in wound healing—led to the choice of WJ-MSCs as a therapeutic agent in our clinical case. Taking into account our results, we conclude that WJ-MSCs have a potential to be an effective adjunct to traditional surgical correction methods, particularly in high-risk patients for whom standard approaches for wound healing are often ineffective. The clinical outcome of such therapy would be complete wound healing, chest wall stabilization, and no signs of recurrent infection even in elderly patients.

However, several limitations may restrict conclusive points of our case report. First, the lack of controlled clinical studies (independent or multicenter) in the use of WJ-MSCs, specifically in chronic sternal wounds, significantly reduces availability of similar or supportive findings. On one hand, the above-mentioned findings on effectiveness of WJ-MSC-based therapy in chronic diabetic wound healing could be in principle translated to other chronic wound states. On the other hand, the thorough assessment of actual beneficial and adverse effects, long-term consequences, and safety issues is nevertheless required. Therefore, our results partially suffer from inability to say conclusively that the described positive effects are solely or predominantly due to the local application of WJ-MSCs. Second, the application of allogenic cell products like WJ-MSCs always poses an issue of the elevated risk of graft-versus-host reaction. In our clinical case, no HLA-DR expression in the WJ-MSCs was detected (≤2% positive cells), which was consistent with background values. This suggests very low risk for immune activation upon injection and complies with biological safety requirements for cell products. However, in certain conditions, like autoimmune defects or immunodeficiency states, special attention is needed to evaluate possible immunological and inflammatory risks, especially if WJ-MSCs are systemically administered. Again, further studies are needed to accumulate a large enough pool of adverse effects (if any) and long-term positive outcomes of WJ-MSC-based therapy.

## 4. Conclusions

The case report illustrates that allogenic Wharton’s jelly mesenchymal stem cells (WJ-MSCs) may facilitate sternal wound healing after CABG. In our case, the use of these cells effectively altered the progression of refractory chronic osteomyelitis in an elderly, high-risk patient (also additionally complicated by a positive COVID-19 status). The two-stage surgical approach, including radical debridement of the infection site and subsequent therapy by WJ-MSCs, not only mitigated and canceled chronic inflammatory process but also significantly accelerated reparative processes in the affected area. Our experience of using WJ-MSCs suggests potential therapeutic benefits for the treatment of complex deep sternal wound infections. Their use can be recommended for wider clinical applications as a safe and effective strategy. However, further prospective studies and clinical series are necessary to clarify the indications, optimize administration regimens, and confirm the long-term efficacy of this approach, thereby integrating it into modern standards of specialized surgical care.

## Figures and Tables

**Table 1:** Comparative characteristics of perinatal MSCs (P-MSCs) and adult MSCs (A-MSCs).

Parameter	P-MSC (Source: Wharton’s Jelly)	A-MSC (Source: Bone Marrow)
Invasiveness of collection	Non-invasive	Highly invasive
Need for regular replenishment	Low, recyclable medical waste	High, needs regular recollection (aspiration under anesthesia)
Proliferative potential	Very high, rapid population doubling	Low, decreases significantly with the age of the donor
Immunogenicity	Extremely low (immunoprivileged status due to low HLA-I/II expression)	Moderate (higher expression of HLA markers compared to P-MSCs)
Risk of tumorigenicity (teratomas)	Negligible (no teratoma formation)	Low (risk of accumulation of age-related genetic mutations)
Ethical limitations	No	Minimal (informed consent for an invasive procedure)
Invasiveness of collection	Non-invasive	Highly invasive

## Data Availability

The original contributions presented in this study are included in the Supplementary Material. Further inquiries can be directed to the corresponding authors.

## Supplementary Materials

The following supporting information can be downloaded at: https://www.mdpi.com/article/10.3390/diseases14010027/s1, Figure S1: Expression of CD73+ and CD90+ markers on mesenchymal stem cells (MSCs) before and after cultivation. Percentage is indicated for double-positive cells; Figure S2: Expression of CD49a+ and CD105+ markers on mesenchymal stem cells (MSCs) before and after cultivation. Percentage is indicated for cells that were positive for CD105 only; Figure S3: Changes in the interleukin profile of mesenchymal stem cells (MSCs) before and after cultivation; Table S1: Growth kinetics and cell count: Analysis within the period from Day 1 to Day 20 [51,52,53].

## Abbreviations

The following abbreviations are used in this manuscript:

A-MSC	“Adult” Mesenchimal Stem Cell
AD-MSC	Adipose Tissue Mesenchimal Stem Cell
BM-MSC	Bone Marrow Mesenchimal Stem Cell
CABG	Coronary Artery Bypass Grafting
CRP	C-Reactive Protein
DSWI	Deep Sternal Wound Infection
ESC	Embryonic Stem Cell
HLA	Human Leukocyte Antigen
LVEF	Left Ventricular Ejection Fraction
MSC	Mesenchimal Stem Cell
P-MSC	Perinatal Mesenchimal Stem Cell
WJ-MSC	Wharton’s jelly Mesenchimal Stem Cell
