# Supplementary material for: Intradermal Application of Allogenic Wharton’s Jelly Mesenchymal Stem Cells for Chronic Post-Thoracotomy Wound in an Elderly Patient After Coronary Artery Bypass Grafting: Clinical Case with Brief Literature Review
Source: Diseases. 2026 Jan 8;14(1):27. doi: 10.3390/diseases14010027 (PMC12839787; doi:10.3390/diseases14010027)
Supplement: Supplementary file 1 [file diseases-14-00027-s001.zip › Supplementary Materials (Figure S1-S3; Table S1).pdf]

SUPPLEMENT DATA

Intradermal application of allogenic Wharton's jelly mesen-chymal stem cells for chronic post-thoracotomy wound in an elderly patient after coronary artery bypass grafting: clinical case with brief literature review

Anastassiya Ganina, Abay Baigenzhin, Elmira Chuvakova, Naizabek Yerzhigit, Anuar Zhunussov, Aizhan Akhaeva, Larissa Kozina, Oleg Lookin, Manarbek Askarov

The process of obtaining an allogeneic cellular product from the umbilical cord

Biomaterial sampling

Only healthy donors who have undergone a preliminary medical and biological examination and screening for infectious diseases were allowed to participate in collecting perinatal tissues. Screening included testing for HIV-1/2, hepatitis B and C viruses, syphilis, cytomegalovirus (CMV), HTLV family viruses, and other clinically significant pathogens. The study was performed in accordance with the principles of the Helsinki Declaration of the World Medical Association «Ethical principles of conducting medical research with human participation» and all donors received written informed voluntary consent before starting labor. The signed document regulated the gratuitous nature of tissue transfer, the anonymization of personal data and the use of biomaterial exclusively for scientific and clinical purposes. The umbilical cord fragment was taken after the baby was born and the umbilical cord was crossed, which completely eliminated the risks to the health of the mother and newborn.

The results of the examination were recorded and stored in accordance with the rules of personal data security. After cesarean section, the sampling procedure was performed in a sterile operating environment in compliance with all the rules of asepsis and antisepsis. The separated umbilical cord was placed in a closed sterile container with DMEM F-12 transport medium with the antibiotic CEF3 and albumin 20%. This prevents microbial contamination and preserves cell viability. To ensure the safety and quality of the biomaterial, it was transported in a specially designed refrigerated container at a strictly controlled temperature in a range of +2 to +8 °C. This ensures that the sample is not subject to temperature fluctuations and mechanical damage. The time of delivery of the material to the laboratory did not exceed 24 hours from the moment of delivery in order to preserve the cellular structures and reduce the risk of damage to the biological material.

Isolation of the WJ-MSC cells

An enzymatic method of cell isolation was used. The method is aimed at gently destroying the extracellular matrix of umbilical cord tissue (primarily Wharton's jelly) to release

mesenchymal stromal cells (WJ-MSCs) and fibroblast-like cells suitable for cultivation and further clinical use. The umbilical cord, delivered at a temperature of +2 to +8°C, was pre-washed in saline solution with antibiotics (100 U/ml of penicillin, 100 μg/ml of streptomycin) to remove blood and other non-cellular remnants. This was carried out in sterile conditions of a laminar flow cabinet of safety class II A. Vessels (two arteries and one vein) were removed from the umbilical cord tissue, after which the gelatinous Wharton's jelly was isolated. After that, the umbilical cord material was cut into fragments about 2-5 mm^3 in size, which ensured uniform action of the enzymes. Combination of the following enzymes was used for enzymatic dissociation: collagenase type 1 + collagenase type 2 (PanEco, Russia), both enzymes were dissolved at the concentration of 0,015% in Ringer's Solution. The solution was prepared immediately before use and filtered through a 0.75 μm sterile filter. The digested tissue fragments were placed in a sterile 50 mL centrifuge tube and placed in a CO_2 incubator with a shaker, thus ensuring gentle stirring at 37°C for 1 hour with visual control for uniform cell dissociation and prevention of enzymatic cell damage.

After 60 min of enzymatic digestion, Albumin of 20% was added to inactivate the enzyme. After adding albumin, after 1-2 minutes, the mixture was filtered through a 75 μm filter. The cells were centrifuged at standard speeds, the cell pellet was washed and seeded into 75 cm^2 culture flasks. The heterogeneous cell population was cultured for 4 weeks in a CO_2 incubator, with passage every 5th day, until a monolayer and a morphologically fibroblast-like structure were achieved.

Safety and quality studies of the cellular product before clinical use

To confirm the safety of the biomedical cell product, a complex of laboratory studies was conducted that meet current regulatory requirements. The assessment included sterility control, screening for the absence of infectious agents, and checking the viability of the cell population.

Microbiological purity and sterility control, aimed at detecting aerobic and anaerobic microorganisms, as well as fungi, were performed in a certified microbiological laboratory in compliance with aseptic rules. The assessment was performed on an automatic analyzer BacT/ALERT 3D 120 Combo (bioMérieux). The 4 mL cell product samples were inoculated into specialized BacT/ALERT PF Plus vials containing a complex nutrient medium and adsorbing polymer granules. Incubation was carried out for 8 days with continuous automatic monitoring. The product was considered sterile (safe) only if there were no signs of microflora growth during the entire observation period.

In parallel, an assessment was carried out for the absence of Mycoplasma spp contamination, which was confirmed by polymerase chain reaction (PCR). A study on vector-borne infections was performed by real-time PCR. The testing panel included key viral pathogens:

HIV-1/2 (HIV), hepatitis B and C viruses (HBV, HCV), human T-lymphotropic virus (HTLV-1/2), cytomegalovirus (CMV) and other herpesviruses. Validated commercial reagent kit ("AmpliSens") was used using internal positive and negative controls. According to the safety protocol, upon receipt of a confirmed positive result, the corresponding sample was immediately excluded from further clinical use.

The pyrogenicity of the drug was assessed by quantification of bacterial endotoxins using the LAL test (Limulus Amebocyte Lysate), which is recognized as the "gold standard" in the biomedical products industry. The assessment was performed using the kinetic chromogenic method on an Endosafe®-PTS™ analyzer (Charles River Laboratories). This technique allows obtaining quantitative results with high sensitivity (up to 0.005 UE/mL) and eliminates the risk of pyrogenic reactions in the recipient. The endotoxin content in the final product was within acceptable limits for parenteral administration.

The functional quality of the product was assessed by calculating the total concentration and proportion of living cells using an automatic CountessII counter (Thermo Fisher Scientific). The population viability of at least 85% was considered the criterion for the suitability for transplantation.

Method of administration of a cellular heterogeneous suspension

The suspension was administered around the perimeter of the wound in a sterile operating room three times with a 1-day interval using a sterile insulin syringe, 1 mL, previously diluted in saline solution. The number of viable cells in 1 mL was as follows: first dose – 5.86×10^6 cells, second dose – 6.28×10^6, third dose – 5.24×10^6 cells. The growth dynamics and morphology of cell cultures are shown in the table (Table S1).

Table S1. Growth kinetics and cell count: Analysis within the period from Day 1 to Day 20.

**Day**	**The growth phase**	**Estimated number of cells (T75)**	**Confluence (%)**	**Biological characteristics and actions**
1	Log Phase / Adaptation	$3,75 \times 10^5$	5-10%	The cells attach to the plastic, change shape from rounded to fusiform. The divisions are singular. Active synthesis of matrix proteins.
2	Early log phase	$6,5 \times 10^6$	15-20%	The beginning of active division. PDT ~24-29 h. Formation of small colonies.

				The cells acquire a typical fibroblast-like morphology.
3	The middle log phase	$1,3 \times 10^6$	35-45%	"Exponential growth. Colonies begin to merge. High metabolic activity (glucose consumption
4	Late log phase	$2,4 \times 10^6$	70-80%	The optimal window for the passage. The cells form a monolayer. A "whirlpool" or "fingerprint" pattern is visualized.
5	Confluence / Plateau	4×10^6	90-100%	Contact braking. A slowdown in growth. The risk of spontaneous differentiation or the beginning of the formation of 3D aggregates in the absence of passage.
6	Passage 1	$3,75 \times 10^6$	5-10%	Transferred to new vials. If left unchecked, the cells thicken, the size of the cytoplasm decreases, and apoptosis or detachment begins.
7	Log phase (P1)	7×10^5	20%	Active proliferation of a new generation. High expression of stem markers (CD90, CD105, CD73).
8-9	Log phase (P+1)	$2,5 \times 10^6$	70-80%	Re-achieving subconfluence. Ready for the next passage.
10	Log phase (P+2)	$3,75 \times 10^5$	5-10%	The third expansion cycle is within 20 days. The cumulative population doubling (CPD) reaches 6-8.
15	Log phase (P+2)	$2,6 \times 10^6$	80%	Stable kinetics. The morphology remains homogeneous (small fusiform cells).
20	Saturation phase	Variable	100%	During prolonged cultivation without sieving (from day 15), the formation of multilayer structures, changes in the pH of the medium, and signs of aging are observed.

The growth kinetics analysis was performed for the T75 vial cultivation model, which is the standard for laboratory expansion. The scenario under consideration involves the use of early

passage cells (P3–P5), since it is at this stage that cells demonstrate maximum proliferative activity and genetic stability.

Formulas for calculating the doubling time (PDT) and cumulative population doubling (CPD) were used to accurately describe the growth. PDT is calculated as:

$$PDT = \frac{\mathrm{T} * \ln(2)}{\ln(N_{\mathrm{h}}) - \ln(\mathrm{N_i})}$$

where T is the cultivation time in hours, $\mathrm{N_h}$ is the number of collected cells, $\mathrm{N_i}$ is the initial number of cells.

An analysis of the literature indicates that WJ-MSC have the shortest PDT among all types of MSCs (24-40 hours), significantly ahead of MSCs of bone marrow (40-70 hours) and adipose tissue (30-50 hours) [53]. This property makes it possible to obtain clinically significant doses of cells (hundreds of millions) in a short time, which is critical for patients with acute conditions [51-52].

Investigation of the effectiveness of the obtained cellular material by methods of flow cytoflowmetry

To assess the phenotypic characteristics and effectiveness of the obtained cell material, an analysis by flow cytofluorometry was performed. The study was aimed at determining the proportion of viable cells and the expression of markers characteristic of mesenchymal stem cells (MSCs). The analysis was performed on a BD Facscalibur flow cytofluorimeter using appropriate isotypic controls and standard antibody panels. The following surface markers were evaluated to identify the cell population: CD73, CD90, CD49a, CD105 (positive MSC markers), CD34, CD14, CD19 (negative markers).

The expression of HLA-DR (MHC class II) molecules on the cell surface was assessed by flow cytometry using fluorescently labeled monoclonal antibodies to HLA-DR. The analysis was performed on viable cells after excluding cell debris and duplicates. The study revealed no HLA-DR expression on the cell surface (≤2% positive cells), consistent with background values. These data indicate the absence of immune activation in the cell cultures, demonstrate their low immunogenicity, and comply with the biological safety requirements for cell products.

The presented graphs showing the expression of CD90 and CD73 (as well as their coexpression) in MSCs show the results of cell material typing before and after cell culture (Figure S1). It should be noted that before cultivation, the main cell population was located in the fluorescence region corresponding to $CD90^{+}/CD73^{+}$ coexpression, which indicates the presence

of a mixed population of MSCs, including both mature and less differentiated progenitor cells. After cultivation, the main cell population shifted to a region with a higher fluorescence intensity for both markers (CD90⁺/CD73⁺), reflecting an increase in the proportion of mature MSCs and standardization of the cell culture phenotype in accordance with the International Society for Cellular Therapy criteria. The results obtained confirmed the phenotype meeting the above-mentioned criteria, and the high purity and viability of the cell population, which indicates the effectiveness and quality of the obtained cell material after the cultivation stage. In addition, Figure S2 shows the flow cytometry plots obtained for cells stained by CD49a and CD105 before and after cultivation. Despite the decrease in the expression of CD49a in culture, there are many new cells appeared as expressing CD105, which is also one of the minimal criteria accordingly to the International Society for Cellular Therapy criteria.

To assess the secretory activity of MSCs, an interleukin status analysis was performed by determining the cytokine level using ELISA on an automatic analyzer «Alisei» (Seac, Italy) in accordance with the instructions of the manufacturer of reagent kits, which made it possible to simultaneously assess the concentrations of both anti-inflammatory and pro-inflammatory factors. The following cytokines were included in the study: IL-6, IL-8, IL-10, IL-1β, TNF-α, TGF-β1.

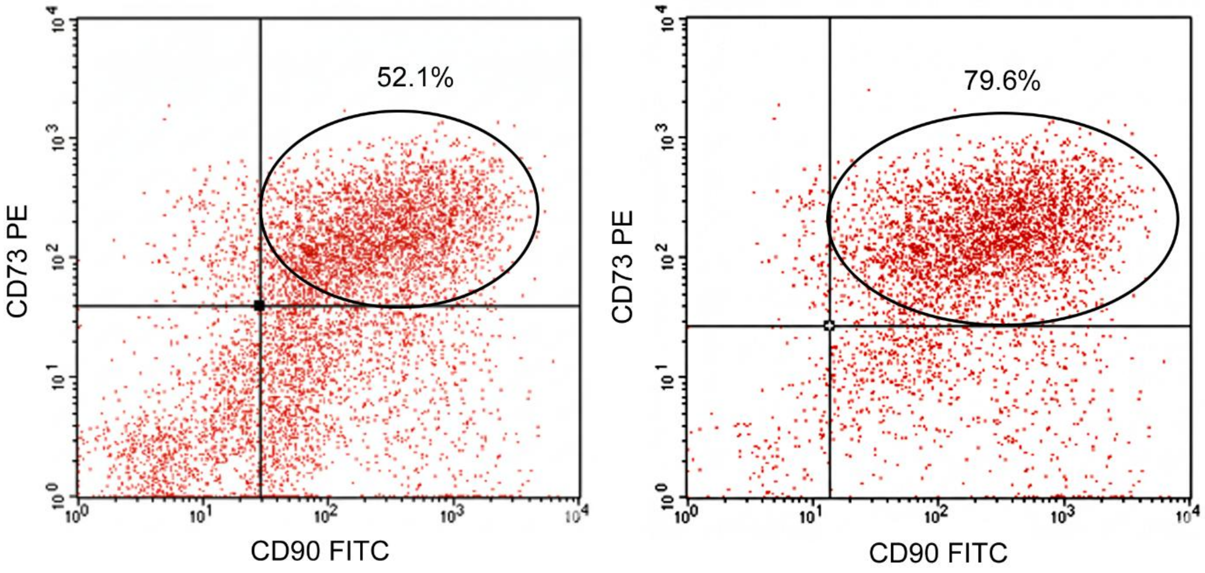

Figure S1. Expression of CD73+ and CD90+ markers on mesenchymal stem cells (MSCs) before and after cultivation. Percentage is indicated for double-positive cells.

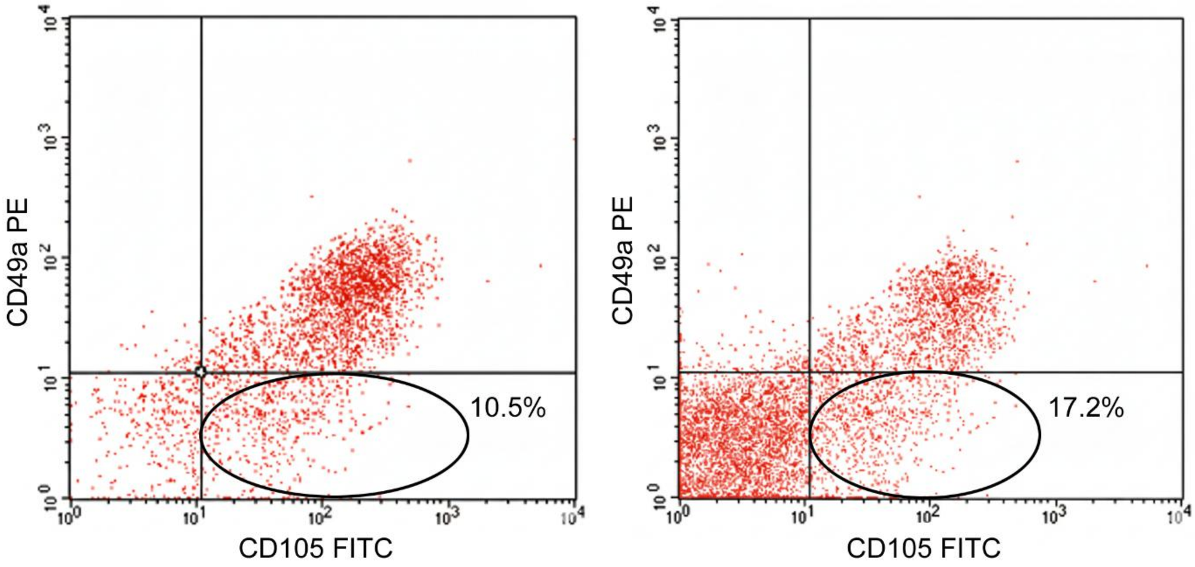

Figure S2. Expression of CD49a+ and CD105+ markers on mesenchymal stem cells (MSCs) before and after cultivation. Percentage is indicated for cells that were positive for CD105 only.

Before cultivation, the cell material was characterized by a mixed cytokine profile with moderately elevated levels of IL-6 and IL-8, which, in our opinion, reflected the response to mechanical stress and adaptation of cells after isolation. Concentrations of anti-inflammatory cytokines IL-10 and TGF-β1 remained low during this period, which corresponded to the early phase of the cellular response and the presence of a heterogeneous population. After cultivation under standard conditions (37°C, 5% CO_2) MSCs formed a stable anti-inflammatory profile characteristic of mature mesenchymal cells, which was confirmed by increased levels of IL-10 and TGF-β1, while reducing concentrations of IL-1β, IL-6, IL-8 and TNF-α (Figure S3).

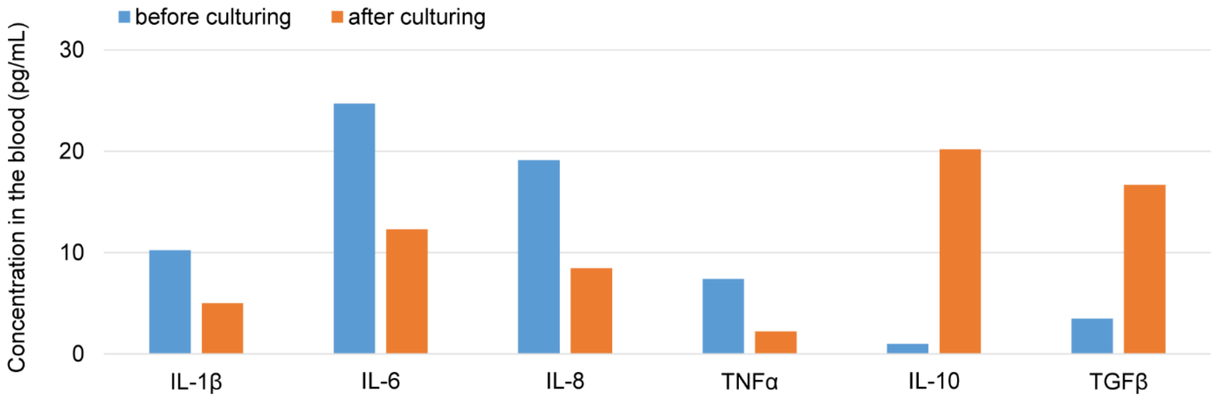

Figure S3. Changes in the interleukin profile of mesenchymal stem cells (MSCs) before and after cultivation.

It is this ratio of cytokines that indicates the restoration of the immunoregulatory and regenerative potential of cells. Thus, the data obtained indicate the transition of the cytokine status of MSCs from a mixed (adaptive-inflammatory) to an anti-inflammatory and immunomodulatory type, which confirms the preservation of the functional properties of the cell product during cultivation.
