# Supplementary figures and images for: Intradermal Application of Allogenic Wharton’s Jelly Mesenchymal Stem Cells for Chronic Post-Thoracotomy Wound in an Elderly Patient After Coronary Artery Bypass Grafting: Clinical Case with Brief Literature Review
Source: Diseases. 2026 Jan 8;14(1):27. doi: 10.3390/diseases14010027 (PMC12839787; doi:10.3390/diseases14010027)

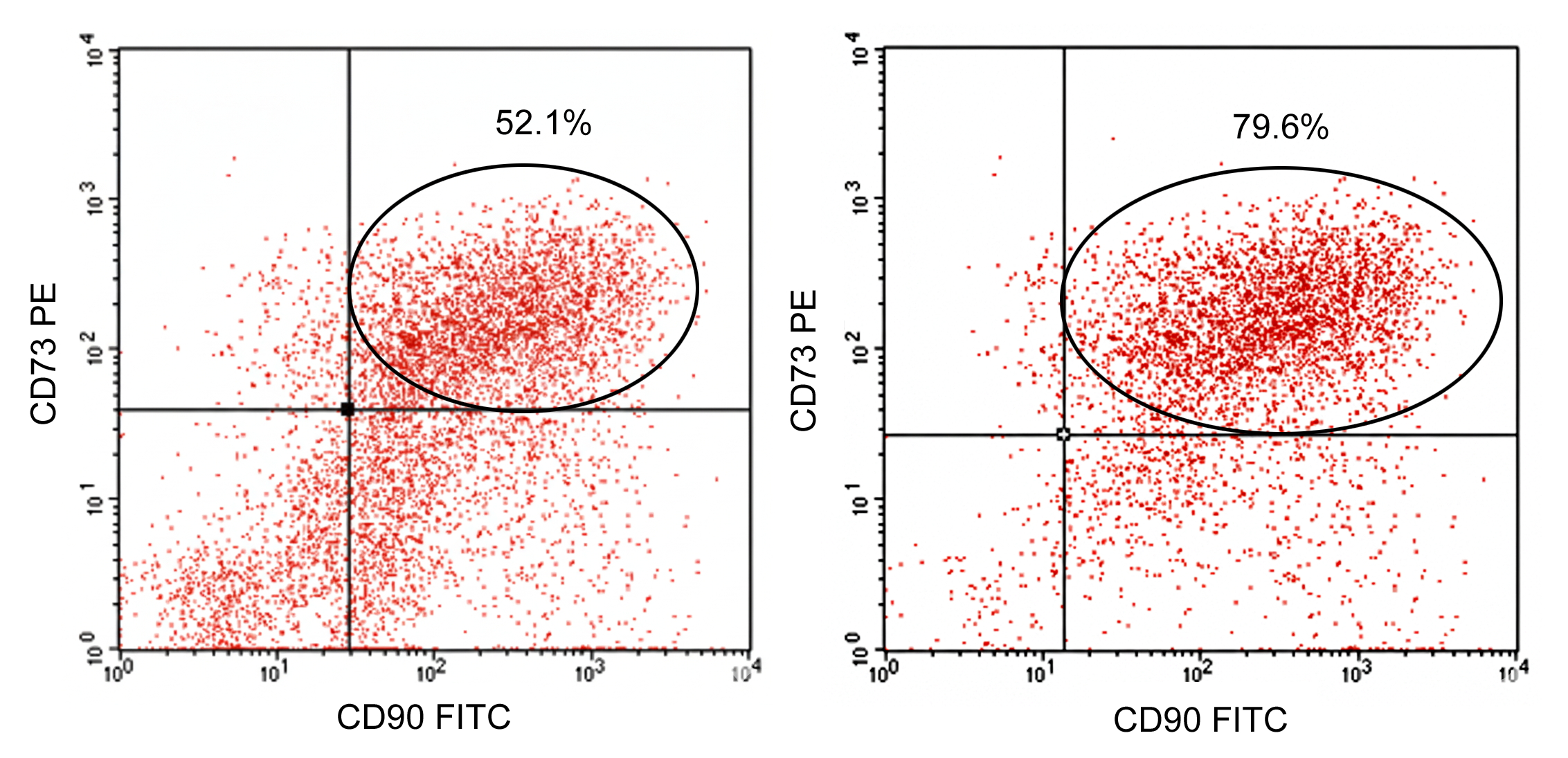

Supplement: Supplementary file 1 [file diseases-14-00027-s001.zip › FigureS1.tif]

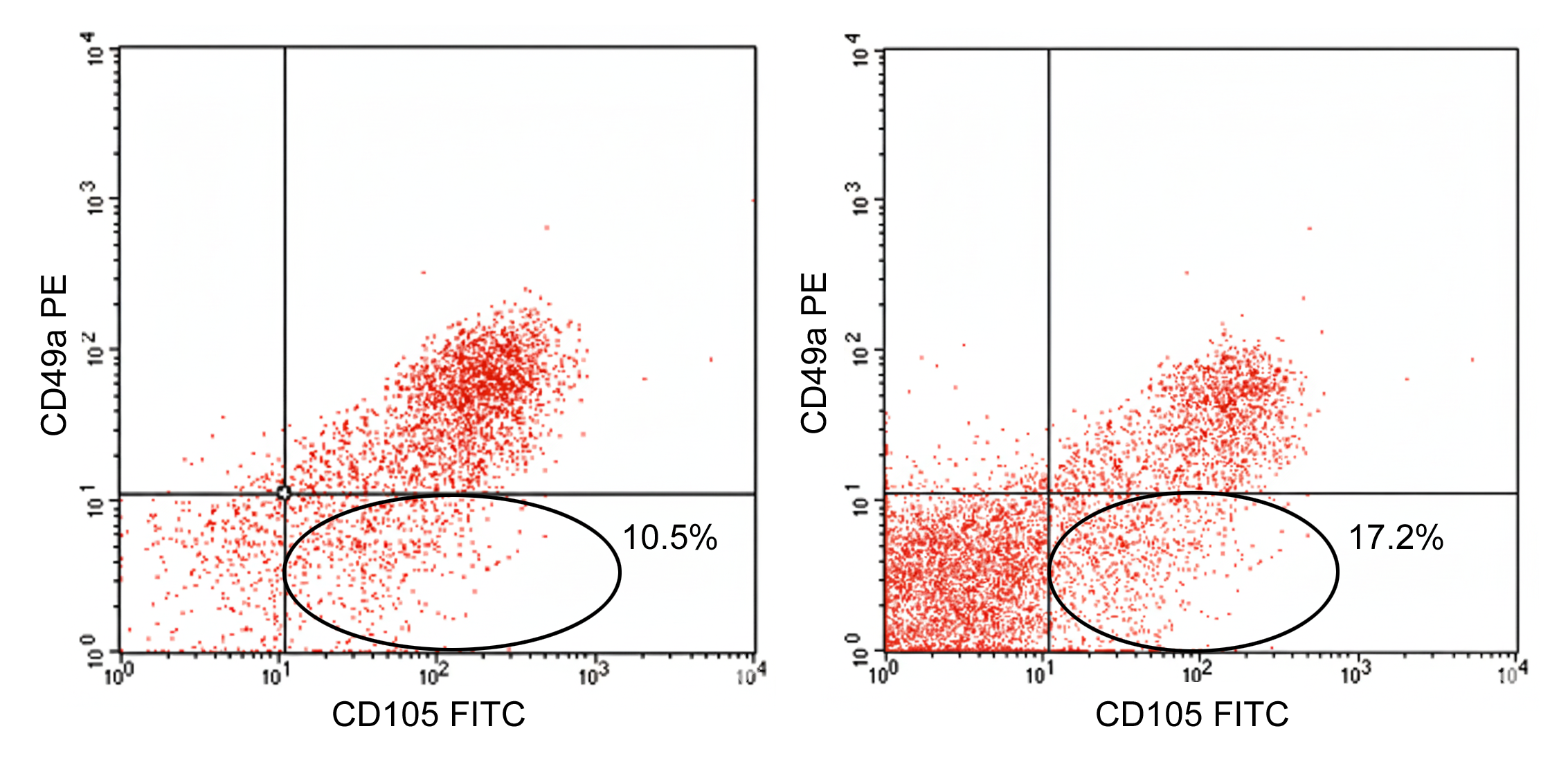

Supplement: Supplementary file 1 [file diseases-14-00027-s001.zip › FigureS2.tif]

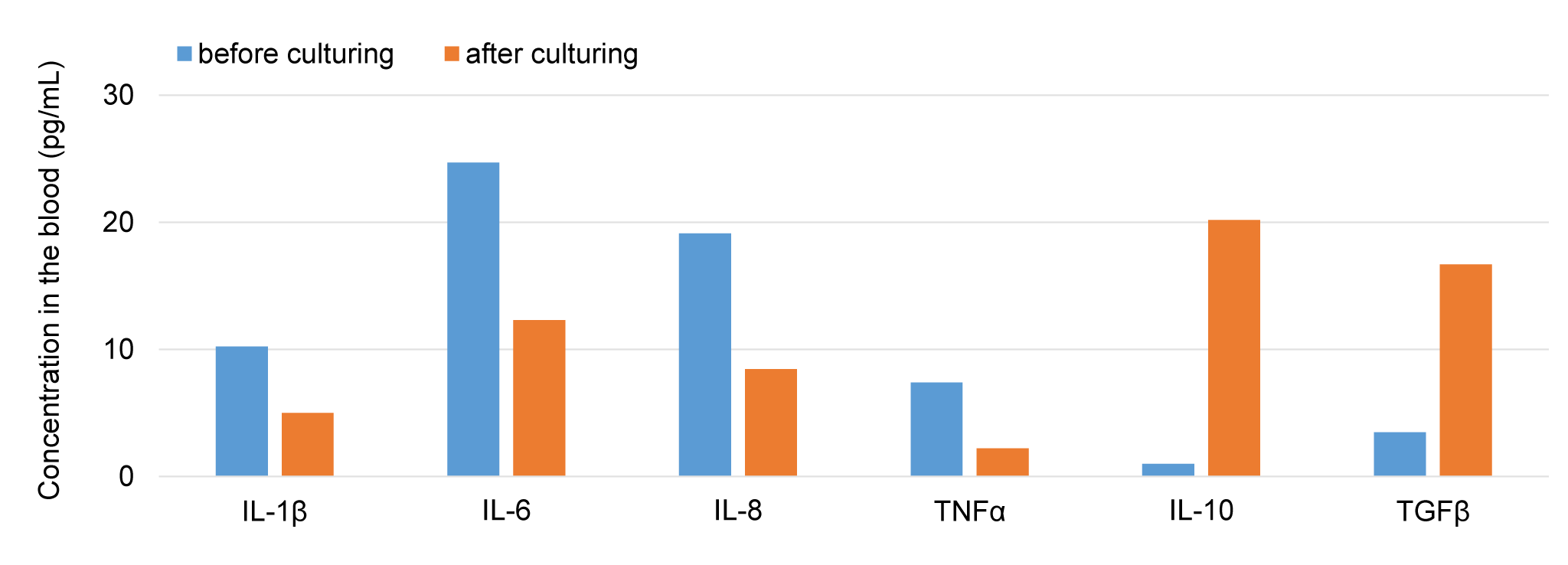

Supplement: Supplementary file 1 [file diseases-14-00027-s001.zip › FigureS3.tif]
